# Bacterial Contaminants and Antibiogram of Ghana Paper Currency Notes in Circulation and Their Associated Health Risks in Asante-Mampong, Ghana

**DOI:** 10.1155/2020/8833757

**Published:** 2020-10-08

**Authors:** Denis Dekugmen Yar

**Affiliations:** Department of Science Education, Faculty of Science and Environment Education, College of Agriculture Education, Asante-Mampong, University of Education Winneba, Winneba, Ghana

## Abstract

Transmission of pathogens through currency notes has become very relevant in today's world due to COVID-19 pandemic. This study profiled microbial flora and their antibiotic activities from Ghana paper currency (GH¢) notes in circulation in Mampong Municipal of Ashanti Region, Ghana. The study employed a cross-sectional design to assess bacterial contaminants and their antibiotic activities from January to May 2019. A total of 70 GH¢ notes consisting of 15 each of GH¢1, GH¢2, and GH¢5; 10 each of GH¢10 and GH¢20; and 5 of GH¢50 were randomly sampled from persons at different shops, canteens, and commercial drivers. The surfaces of each GH¢ note were gently swabbed, and tenfold serial dilutions made were inoculated on plate count agar (PCA), MacConkey agar, mannitol salt agar, and deoxycholate citrate agar. The study used appropriate laboratory and biochemical tests for bacterial identification. SPSS-IBM version 16.0 was used to analyze the data. Of the 70 GH¢ notes studied, 97.1% were contaminated with one or more bacterial isolates. Mean counts on PCA ranged between 3.2 cfu/ml × 10^5^ and 4.7 cfu/ml × 10^5^ on GH¢ notes. Of 124 bacteria isolated, 34 (27.4%), 30 (24.2%), 22 (17.7%), 17 (13.7%), 13 (10.5%), and 8 (6.5%) were from GH¢1, GH¢2, GH¢10, GH¢5, GH¢20, and GH¢50, respectively (*p* < 0.05). Bacterial isolates were *Escherichia coli* (28.23%), *Staphylococcus aureus* (16.94%), coagulase-negative *Staphylococcus* (16.13%), *Klebsiella* species (11.29%), *Salmonella* species (9.68%), *Shigella* species (8.87%), *Pseudomonas aeruginosa* (5.65%), and *Proteus* species (3.23%). GH¢ notes had 25.81%, 20.16%, 19.35%, 17.74%, and 16.94% from meat shops, commercial drivers, canteens, grocery shops, and vegetable shops, respectively. All bacteria were 100% resistant to erythromycin, 87.5% to tetracycline, chloramphenicol, and cotrimoxazole, 75% to vancomycin, while 87.50% sensitive to amikacin. The GH¢ notes were heavily colonized with potential pathogens, which are resistant to most commonly used antibiotics and could pose a health threat to users during commercial transactions.

## 1. Introduction

Globally, paper currency notes are widely used to exchange for goods and services [1–3]. Users often contaminate these notes with several microflora including viruses, fungi, protozoan, and bacteria via unhygienic conditions and habits [1, 4–6]. Some of these practices and habits include nose-picking, coughing and sneezing on palms, applying saliva on fingers to aid notes counting, improper hand washing after using toilets, and storage of paper notes on contaminated surfaces [3, 7].

Historically, money was strongly linked to fatal infections like the “Black Death” or bubonic and pneumonic plague pandemics [8–10]. Currency notes, therefore, serve as fomites and vehicles for the transfer of microflora from one user to another [11–13]. Infection with pathogens through contact with fomites is widespread and more serious in the era of COVID-19 pandemic. Individuals get infected by touching objects or surfaces contaminated with pathogenic microbes while touching the eyes, nose, or mouth [14].

Infections caused by microflorae on currency notes are largely bacterial and many of them defied treatment with antibiotics [15–19]. Resistance to antibiotic treatment by some of these bacteria had claimed millions of lives despite huge investments and efforts to decrease the predicaments [20, 21]. This has a dire global health consequence and is incumbent to check the disease transmission and antibiotic resistance via fomites [12, 22]. Global surveillance showed there is a widespread antibiotics resistance [18, 23, 24] including drug-resistant *Streptococcus pneumoniae*, vancomycin-resistant *Staphylococcus aureus*, methicillin-resistant *Staphylococcus aureus*, and multidrug-resistant *Pseudomonas aeruginosa* [17, 25, 26].

In Ghana, Mobile money (M-money) and Electronic commerce (E-commerce) payments for goods and services in the informal sector is very low [27, 28]. There is, however, a gradual use (4.8%) of M-money and E-commerce due to some perceived risk issues and service charges [29]. Other studies showed that the majority of participants preferred cash instead of M-money to pay for goods and services [30, 31]. The Ghana paper currency (GH¢) notes are constantly passed on from one user to another with dirty hands, squeezed, mutilated, and kept on contaminated surfaces [32].

Earlier studies also reported that the GH¢ notes were heavily colonized by microflorae [1, 33], with some being pathogenic [32]. These notes, therefore, carried pathogenic bacteria that can infect handlers [34] and are resistant to antibiotics [35]. In Ghana, money is the most often and widely passed item from one user to another for commercial transactions. Meanwhile, pathogenic bacteria colonized our hands although adherence to effective hand washing is not observed by most Ghanaians [36]. Adherence to COVID-19 protocols among Ghanaians was found to be difficult. The mantra “to wash your hands regularly with soap and water or clean with alcohol-based hand rub” cannot be overemphasized [14].

Infections with pathogens via fecal-oral route from fomites are rampant [37]. There is, however, poor adherence to effective hand washing among many food vendors in Ghana [38] although cooked food is sometimes served with bare hands [39], thus putting customers at risk of foodborne infections [40]. There are several studies conducted on microbial contamination on GH¢ notes mainly in major cities in Ghana. There is, however, paucity of empirical data on bacteria and their antibiotic profile on GH¢ notes in peri-urban Ghana. This study examined the microbial flora and their antibiotic activities from the Ghana paper currency notes.

## 2. Methodology

### 2.1. Study Area

The study area was in the Mampong Municipal of Ashanti Region, Ghana. It is one of the twenty-seven administrative districts located north of the region and shares boundaries with Atebubu-Amantin Municipal, Sekyere East, Afigya-Sekyere, and Ejura-Sekyeredumase to the north, east, south, and west [41]. The municipal is within longitudes 0.05 degrees and 1.30 degrees west and latitudes 6.55 degrees and 7.30 degrees north, covering a total land area of 2346 km^2^. The municipal capital Mampong is about 57 km from the regional capital Kumasi. The municipal population is about 88,051, out of which 42,653 are males and 45,398 are females. Agriculture is the main economic activity within the municipal and employs about 67.3% of the entire labor force. The climate and topography of the area are conducive for agriculture [42, 43].

### 2.2. Study Design

This study employed a descriptive cross-sectional design to assess microflora contamination on Ghana paper currency (GH¢) notes and their antibiotic activities in Mampong Municipal from January to May 2019.

### 2.3. Sample Size and Sampling

The currency notes included GH¢1, GH¢2, GH¢5, GH¢10, GH¢20, and GH¢50, which are the most widely circulated denominations. A total of 70 GH¢ notes made up of 15 GH¢1, 15 GH¢2, 15 GH¢5, 10 GH¢ 10 GH¢20, and 5 GH¢50 were randomly sampled from different sources in the Mampong township. This study sampled only GH¢ introduced by the Bank of Ghana in 2007. The numbers of each GH¢ note were sampled based on the frequency of usage and volume in circulation.

### 2.4. Sample Collection Procedures

The study used aseptic techniques to collect each GH¢ note into a separate plastic envelope bag and sealed. These notes were collected from grocery shops, canteens, commercial drivers, butchers outlets, and vegetable shops. The notes were obtained either by exchanging fresh banknotes for old notes or through a commercial activity. The study used a week to sample each denomination to improve efficiency.

### 2.5. Laboratory Methods and Analysis

#### 2.5.1. Media Used for Culture

The media used were the products of Oxoid Limited, Basingstoke Hampshire, England. The study adopted the Oxoid standard protocol for media preparation except for selenite F broth. The media included plate count agar (PCA), MacConkey agar (MCA), mannitol salt agar (MSA) and deoxycholate citrate agar (DCA), Simmons citrate agar, triple sugar iron agar (TSI), selenite broth, and peptone water. Reagents were Kovacs and Remel BactiDrop Oxidase. PCA, MCA, DCA, and MSA were the media used for the total viable count, Gram-negative bacteria, S*almonella-Shigella*, and *Staphylococcus* isolation in that order. Mueller–Hinton agar (MHA) was the medium used for the antimicrobial susceptibility test.

#### 2.5.2. Sample Processing

Each GH¢ note was given a unique identifier. Both surfaces of the note were gently swabbed with sterile cotton moistened with sterile buffered peptone water (BPW). The swabs in their respective tubes with 1 ml sterile BPW was then vortexed to get a uniform suspension. The study made tenfold serial dilutions of each suspension for cultivation and identification of microbial contaminants. The GH¢ notes swabbed for the study were later cleaned with MAK SWAB (alcohol swab).

#### 2.5.3. Cultivation and Enumeration

The study used 0.1 ml of each dilution, and each dilution was inoculated into the right media and incubated at 37°C for 12–48 hours. The culture plates were then examined for growth and morphologic characteristics based on standard microbiological methods. Discrete colonies were subcultured on Nutrient Agar for biochemical analysis and Gram's staining technique [44]. Electronic colony counter was then used to count the total viable colonies, and the average counts were expressed in cfu/ml [44].

#### 2.5.4. Identification of Isolates

The study identified bacterial isolates using standard microbiological methods [45]. Gram's staining, colony morphology, and suitable biochemical tests were the methods used to identify bacterial isolates. For Gram-positive cocci bacteria with purple round shapes, catalase and coagulase tests differentiated staphylococci (catalase-positive) from streptococci (catalase-negative). Isolates of Gram-negative rods on MCA were further grouped into lactose and nonlactose fermenters. These isolates were then inoculated into TSI, and indole test, citrate test, etc., were performed to aid identification [44].

*(1) Salmonella and Shigella species*. Colonies from samples pre-enriched in Selenite F Broth (DifcoTM) plated onto DCA with pale or colourless colonies with or without a black spot in the middle was suggestive of *Salmonella*, while colonies with pink zone indicates *Shigella* [44].

*(2) Staphylococcus species*. Colonies on MSA plates with yellow colonies with halo zone and colonies with pink with reddish-purple zones indicated *Staphylococcus aureus* and *Staphylococcus epidermidis*. Coagulase test differentiates between *Staphylococcus aureus* (coagulase-positive) and *Staphylococcus epidermidis* (coagulase-negative *Staphylococcus* (CNS)) [44].

*(3) Other Gram-negative isolates*. All Gram-negative bacteria from MCA were subsequently inoculated in TSI and were incubated. Growth with acidic butt, acidic slant, and gas production without hydrogen sulphide (H₂S) indicated either *E. coli* or *Klebsiella* species. The two isolates were further identified by indole and citrate tests. Red ring formation on the surface of indole indicates *E. coli.* Blue colour change of citrate after incubation confirms *Klebsiella* species. An acidic butt, acidic slant, and gas production with H_2_S indicates *Proteus* spp. Alkaline butt, alkaline slant, no gas, and H_2_S production indicates *Pseudomonas aeruginosa* [44].

### 2.6. Antibiotic Susceptibility Testing (AST)

Agar diffusion technique on Mueller–Hinton agar (Kirby–Bauer modified disc diffusion technique) according to CLSI guidelines [46, 47] determined the antibiotic susceptibility. The inhibition zone standards for antimicrobial susceptibility were from tables of interpretative zone diameters of Clinical and Laboratory Standards Institute [48]. The study tested 10 antibiotic discs of the most commonly used drugs to treat human and animal infections caused by bacteria. These included erythromycin (ERY) (5 *µ*g), ciprofloxacin (CIP) (5 *µ*g), gentamycin (GEN) (10 *µ*g), ampicillin (AMP) (10 *µ*g), amoxicillin (AMX) (5 *µ*g), vancomycin (VAN) (30 *µ*g), tetracycline (TET) (30 *µ*g), chloramphenicol (CHL) (30 *µ*g), amikacin (AMK) (30 *µ*g), and cotrimoxazole (COT) (25 *µ*g) [49, 50].

### 2.7. Statistical Analysis

Raw data from microbial analysis were then entered into a Microsoft Excel 2007 spreadsheet and transformed the counts into log_10_ for normal distribution. The data were later exported into Statistical Package for Social Sciences (SPSS-IBM) version 16.0 software and analyzed using descriptive statistics and presented as tables. The analysis compared sources of currency notes using *t*-test at 95% confidence interval (*p* ≤ 0.05 and *p* ≥ 0.05), and comparison of means was done using Tukey–Kramer (Tukey's *W*) multiple comparison analysis.

### 2.8. Ethical Consideration

Formal permission was sought from the municipal authorities in Mampong. This study did not collect personal data from study participants. The study, therefore, obtained only verbal consent and approval from participants before sample collection.

### 2.9. Assumptions

This study assumed that canteens, vegetable shops, butchers outlets, grocery shops, and commercial drivers were avenues in which currency notes are often exchanged and passed on from one user to another on daily basis.

## 3. Results and Discussion

### 3.1. Results

Out of 70 GH¢ notes examined, 68 (97.1%) had bacteria contaminants.  Table 1 shows mean total bacterial counts from different denominations on different media. Mean counts ranged from 3.2 cfu/ml × 10^5^ to 4.7 cfu/ml × 10^5^ on PCA, 1.7 cfu/ml × 10^4^ to 8.2 cfu/ml × 10^4^ on DCA, 1.2 cfu/ml × 10^5^ to 2.4 cfu/ml × 10^5^ on MCA, and 1.2 cfu/ml × 10^5^ to 2.7 cfu/ml × 10^5^ on MSA.

Table 2 shows distribution of bacteria isolated from five different sources. Out of 124 bacteria isolated, meat shops had 32 (25.8%), 25 (20.2%) among commercial drivers, 24 (19.4%) in canteens, 22 (17.7%) in grocery shops, and 21 (16.9) in vegetable shops. Isolates were *Escherichia coli* (28.2%), *Staphylococcus aureus* (16.9%), CNS (16.9%), *Klebsiella* species (11.3%), *Salmonella* species (9.7%), *Shigella* species (8.9%), *Pseudomonas aeruginosa* (5.7%), and *Proteus* species (3.2%).

Table 3 shows prevalence of bacterial isolates on different GH¢ denominations. Out of 124 isolates, 34 (27.4%), 30 (24.2%), 22 (17.7%), 17 (13.7%), 13 (10.5), and 8 (6.5%) were from GH¢1, GH¢2, GH¢10, GH¢5, GH¢20, and GH¢50, respectively (*p* < 0.05).

Table 4 shows antimicrobial susceptibility patterns of bacteria isolated. All bacteria were 100% resistant to erythromycin, 87.5% to tetracycline, chloramphenicol, and cotrimoxazole, 75% resistant to vancomycin, 62.5% to ciprofloxacin and ampicillin, 50.0% to amoxicillin and ampicillin, while 87.50% sensitive to amikacin.

## 4. Discussion

In this study, nearly all GH¢ notes sampled had bacterial contaminants. This finding was very similar to earlier studies conducted in Ghana [51, 52], Saudi Arabia [53], and the United States of America [54]. Similar findings were also recorded on Iraqi currency [55], South African banknotes [56], Nepal banknotes [57], and Nigerian banknotes [58]. GH¢ notes are largely used to exchange for goods and services on cash basis in the country [30, 31] with limited use of M-money and E-commence [27, 28]. The low usage of M-money and E-commence could contribute to the high microbial contamination of the GH¢ notes.

This study reported high mean bacteria loads on GH¢ notes than similar studies conducted in Ghana [35, 52] and akin to earlier studies in Nigeria [15, 58, 59]. Other studies elsewhere reported high microbial loads on different currency notes that agreed with the finding in this study [60–62]. This study also reported mean counts for coliforms, *Staphylococcus* species, and *Shigella-Salmonella* species on GH¢ notes much higher than those in a similar study on the Nigerian naira [59] and other currencies [60–62]. The high bacterial load in this study could be linked to the rampant abused of the GH¢ notes especially the lower denominations which are often squeezed, mutilated, mishandled, and kept in unhygienic surfaces.

The findings in this study further revealed that GH¢ notes of lower denominations were significantly more contaminated than higher notes, confirming earlier studies in Ghana and Nigeria [35, 63, 64]. Ghanaians are notorious at abusing and mishandling GH¢ notes with careless abundance and impunity. There seems to be little regard for the lower denominations, and this may be linked to the high bacterial loads on the lower GH¢ notes. Moreover, lower GH¢ notes are frequently passed on from one user to another in diverse economic and social activities with unclean hands, further contributing to the high contaminations [64]. Meanwhile, higher GH¢ notes are often saved in banks, more respected, and less frequently handled.

This study also reported eight different bacterial species, of which *S. aureus* was predominant on GH¢ notes. Previous studies recovered similar microbes from GH¢ notes, of which CNS was most prevalent [35, 63, 64]. Money is the most handled and circulated item among the public making it prone to microbial contamination with a potential to cause diseases to handlers. In this study, *Escherichia coli*, *Salmonella* spp., and *Shigella* spp. were pathogens recovered from GH¢ [65]. These fecal bacteria on GH¢ are an indication of lack of effective hand washing after using washrooms [36, 66] and unhygienic practices among users [67].

In this study, there was no link between sources of GH¢ notes and microbial contamination. GH¢ notes collected from meat shops, commercial drivers, and canteens recorded the highest numbers of bacterial isolates. GH¢ notes from meat shops were likely to be contaminated with blood, which is a good medium for bacterial growth [44, 64]. Commercial drivers often exchanged notes with passengers with unclean hands increasing the rate of contamination [1, 4, 5, 39]. Canteens are often patronized by many people where GH¢ notes are exchanged in minutes from one user to another with dirty hands [68]. GH¢ notes from canteens had all species of bacteria isolated, an indication that food vendors' hands possibly carried fecal coliforms and could lead to foodborne illnesses [39, 69, 70]. Customers with unclean hands could further increase their risk of contracting food-related diseases [71]. The prevalence of bacterial contamination was least on GH¢ notes collected from vegetable shops although vegetables are known to carry microbes [72, 73].

This study revealed that most bacteria isolated from the GH¢ notes were resistant to the widely used antibiotics [18, 74]. This has public health implications since these bacteria are pathogens that could easily infect users and would fail treatment with these antibiotics [75]. Moreover, many of these isolates were resistant to multiple antibiotics, which is a threat to public health in Ghana [76]. However, amikacin was the most effective antibiotic against these bacterial isolates on the GH¢ notes. An earlier study reported similar findings in Ghana [51] and other parts of the world [77]. There is an increasing threat of antibiotic resistance due to excessive usage without prescriptions from a pharmacist [78, 79].

This study has demonstrated that the GH¢ notes are heavily contaminated with potential bacterial isolates that are highly resistant to the most widely used antibiotics and are a threat to public health. The GH¢ notes are overly abused with dirty and unhygienic hands contaminating them with pathogenic bacteria with the potential of infecting handlers. It is highly recommended that E-commerce and M-money should be strongly promoted to save the GH¢ notes from abuse and deterioration and the users from harm. The wake of COVID-19 is a testimony for the need to limit or avoid directly handling currency notes for the public good.

### 4.1. Limitations of the Study

The study met some difficulties obtaining GH¢ notes from the sources. The research assistants bought goods from shops to get the required denominations for the study. Some sources also suspected the team of fraud and trading in fake currencies and were very reluctant.

### 4.2. Conclusion and Recommendation

This study has demonstrated that the GH¢ notes are heavily contaminated with potentially pathogenic bacteria that are highly resistant to the most widely used antibiotics and are a threat to public health. The use of E-commerce and M-money is commendable to use for goods and services to minimize the abuse and deterioration of the GH¢ notes.

## Figures and Tables

**Table 1:** Mean microbial counts on different culture media (cfu/ml).

Media	PCA (cfu/ml)		DCA (cfu/ml)		MCA (cfu/ml)		MSA (cfu/ml)	
Currency	Mean	Log	Mean	Log	Mean	Log	Mean	Log
GH⊄1	4.7 × 10^5^	5.67	8.2 × 10^4^	4.91	1.9 × 10^5^	5.28	2.7 × 10^5^	5.43
GH⊄2	4.2 × 10^5^	5.62	6.0 × 10^4^	4.78	1.2 × 10^5^	5.08	2.4 × 10^5^	5.38
GH⊄5	3.8 × 10^5^	5.58	3.1 × 10^4^	4.49	1.9 × 10^5^	5.27	2.2 × 10^5^	5.34
GH⊄10	4.1 × 10^5^	5.61	4.4 × 10^4^	4.64	2.4 × 10^5^	5.38	2.1 × 10^5^	5.32
GH⊄20	4.0 × 10^5^	5.6	1.4 × 10^4^	4.15	2.0 × 10^5^	5.30	2.0 × 10^5^	5.30
GH⊄50	3.2 × 10^5^	5.51	1.7 × 10^4^	4.23	1.5 × 10^5^	5.18	1.2 × 10^5^	5.08

PCA, plate count agar; DCA, deoxycholate citrate agar; MCA, MacConkey agar; MSA, mannitol salt agar.

**Table 2:** Distribution of bacterial isolates according to sources of currency notes.

Currency note sources bacterial isolates	Grocery shops	Meat shops	Vegetable shops	Drivers	Canteens	Total (%)
*Escherichia coli*	7	8	7	7	6	35 (28.2)
*Salmonella* species	2	3	2	2	3	12 (9.7)
*Shigella* species	2	4	1	1	3	11 (8.9)
*Proteus* species	0	2	0	1	1	4 (3.2)
*Pseudomonas aeruginosa*	1	2	1	2	1	7 (5.7)
*Klebsiella* species	2	2	2	3	3	12 (9.7)
CN*-Staphylococcus*	3	5	4	5	4	21 (16.9)
*Staphylococcus aureus*	5	6	4	4	3	22 (17.7)
Total (%)	22 (17.7)	32 (25.8)	21 (16.9)	25 (20.2)	24 (19.4)	124 (100)

**Table 3:** Distribution of bacterial isolates based on the currency notes denomination.

Isolated bacteria	GH⊄1	GH⊄2	GH⊄5	GH⊄10	GH⊄20	GH⊄50	Total (%)
*Proteus* species	0	2	1	1	0	0	4 (3.2)
*Pseudomonas aeruginosa*	1	2	1	2	1	0	7 (5.7)
*Shigella* species	4	3	2	2	0	0	11 (8.9)
*Salmonella* species	5	4	0	2	1	0	12 (9.7)
*Klebsiella* species	4	4	1	2	1	0	12 (9.7)
CN *Staphylococcus*	4	4	3	4	3	3	21 (16.9)
*Staphylococcus aureus*	7	3	4	5	2	1	22 (17.2)
*Escherichia coli*	9	8	5	4	5	4	35 (28.2)
Total (%)	34 (27.4)	30 (24.2)	17 (13.7)	22 (17.7)	13 (10.5)	8 (6.5)	124 (100)

**Table 4:** Antibiotic sensitivity patterns of bacterial isolates from Ghanaian currency paper notes.

Isolated bacteria	ERY	CIP	GEN	AMP	AMX	VAN	TET	CHL	AMK	COT
*Escherichia coli*	R	R	R	S	R	I	R	R	S	R
*Salmonella* species	R	R	R	S	S	S	R	R	S	R
*Shigella* species	R	R	S	R	R	R	R	R	S	R
*Proteus* species	R	R	S	R	S	R	R	S	S	R
*Pseudomonas aeruginosa*	R	S	R	R	S	R	R	R	I	R
*Klebsiella* species	R	S	R	S	S	R	I	R	S	R
CN *Staphylococcus*	R	R	S	R	R	R	R	R	S	R
*Staphylococcus aureus*	R	S	S	R	R	R	R	R	S	S
% that are resistant (R)	100	62.5	50.0	62.5	50.0	75.0	87.5	87.5	0.0	87.5
% that are susceptible (S)	0.00	37.5	50.0	37.5	50.0	12.5	0.0	12.5	87.5	12.5
% that are intermediate (I)	0.00	0.0	0.0	0.0	0.0	12.5	12.5	0.0	12.5	0.0

ERY, erythromycin; CIP, ciprofloxacin; GEN, gentamicin; AMP, ampicillin; AMX, amoxicillin; VAN, vancomycin; TET, tetracycline; CHL, chloramphenicol; AMK, amikacin; COT, cotrimoxazole.

## Data Availability

All data have been included in the manuscript.
